# Adaptive Multi-Model Hierarchical Federated Learning for Robust IoT Intrusion Detection

**DOI:** 10.3390/s26103198

**Published:** 2026-05-19

**Authors:** Shahid Latif, Djamel Djenouri

**Affiliations:** School of Computing and Creative Technologies, University of the West of England, Bristol BS16 1QY, UK; djamel.djenouri@uwe.ac.uk

**Keywords:** cybersecurity, federated learning, intrusion detection, adversarial robustness

## Abstract

The rapid growth of the Internet of Things (IoT) has introduced significant cybersecurity challenges in highly distributed, heterogeneous, and privacy-sensitive environments. Traditional centralized intrusion detection approaches and conventional federated learning (FL) frameworks, which rely on single-model aggregation, are often inadequate in the presence of extreme non-IID data and adversarial conditions. This study proposes an Adaptive Multi-Model Hierarchical Federated Learning (AMM-HFL) framework for robust IoT intrusion detection. The framework operates across client, edge, and cloud tiers and introduces a unified integration of similarity-aware clustering, multi-model aggregation, and dynamic client-side model selection. Unlike existing hierarchical FL approaches, AMM-HFL maintains multiple global models, enabling adaptive personalization and improved representation of heterogeneous data distributions. At the edge level, model updates are clustered to isolate anomalous contributions, while the cloud performs meta-aggregation to refine diverse model representations. Experimental evaluation on the IDSIoT2024 dataset demonstrates detection accuracy up to 96.83–97.54% under IID and 95.64–97.52% under non-IID conditions, while maintaining low computational and cryptographic overhead.

## 1. Introduction

The rapid growth of the Internet of Things (IoT) has led to highly distributed and heterogeneous environments, in which resource-constrained devices continuously generate sensitive data across diverse applications, including healthcare, smart infrastructure, and industrial systems [1,2,3]. This large-scale connectivity increases IoT networks’ exposure to cyber threats, including distributed attacks, data manipulation, and stealthy intrusions, thereby necessitating advanced intrusion detection mechanisms that operate in decentralized, privacy-sensitive settings [4,5,6]. Federated Learning (FL) has emerged as a promising paradigm that enables collaborative model training without sharing raw data, preserving privacy while leveraging distributed intelligence [7,8,9]. However, conventional FL architectures rely on centralized aggregation at the cloud, leading to communication bottlenecks, scalability limitations, and increased vulnerability to adversarial manipulation in large-scale IoT deployments [10,11]. To address these challenges, a two-tier hierarchical FL architecture was introduced in our prior work [12]. This study [12] incorporated edge servers as intermediate aggregators and lightweight cryptographic mechanisms to ensure secure and efficient communication.

Despite these advancements, significant challenges remain in realistic IoT cybersecurity scenarios. IoT data distributions are inherently non-independent and imbalanced, where individual devices observe limited subsets of traffic patterns and attack types, resulting in extreme non-IID conditions that degrade the performance of single global models [13,14]. Furthermore, existing aggregation strategies, including those used in hierarchical FL, rely primarily on averaging-based mechanisms, which are insufficient to defend against sophisticated adversarial behaviors such as stealthy backdoor attacks and adaptive model poisoning [15,16]. Moreover, existing clustering-based or hierarchical FL methods typically operate within a single global model paradigm, limiting their ability to capture heterogeneous data distributions and support client-level adaptation. Although lightweight cryptography ensures the secure transmission of updates, it does not guarantee the trustworthiness of aggregated models in the presence of malicious clients. In addition, prior studies are often evaluated on generic datasets, which limits their applicability to IDS characterized by high-dimensional tabular data, class imbalance, and evolving attack patterns.

To overcome these limitations, an adaptive multi-model hierarchical FL (AMM-HFL) framework is introduced, which departs from conventional single-model aggregation by jointly integrating hierarchical learning, similarity-aware clustering, and multi-model representation within a unified architecture. The framework operates across client, edge, and cloud tiers. At the client level, local models are trained with a deep neural network, and updates are securely transmitted via lightweight cryptography. At the edge level, decrypted updates are expressed as deviations from the global model and processed using a similarity-aware clustering mechanism, in which consistent updates are grouped, and anomalous updates are isolated or excluded based on statistical thresholds. Each cluster produces an intermediate model, forming multiple candidate representations. At the cloud level, these models undergo meta-aggregation to merge similar clusters while preserving diversity, resulting in a refined set of global models. These models are redistributed to clients, where each client dynamically selects the most suitable model based on local loss evaluation. This integration of hierarchical aggregation, similarity-driven clustering, and multi-model learning provides an implicit defense against adversarial manipulation while improving performance under extreme non-IID conditions.

The main contributions of this work are summarized as follows:

- **Adaptive hierarchical federated learning framework**: An enhanced edge–cloud FL architecture is developed by integrating similarity-aware aggregation to improve robustness and scalability under extreme non-IID IoT environments.
- **Multi-model learning with dynamic client adaptation:** A multi-model aggregation strategy is introduced, enabling the generation of multiple candidate models and dynamic client-side model selection based on local performance.
- **Robust and secure aggregation mechanism:** A unified approach combining lightweight cryptography with clustering-based statistical anomaly isolation is designed to mitigate naive and stealthy poisoning attacks while maintaining low computational and communication overhead.

The remainder of this paper is organized as follows. Section 2 reviews the related work on hierarchical federated learning and IoT intrusion detection. Section 3 presents the proposed framework, including the system architecture, secure communication mechanisms, and aggregation strategies. Section 4 describes the experimental setup and evaluates the performance of the proposed approach under various data distributions and adversarial scenarios. Finally, Section 5 concludes the paper and outlines potential directions for future research.

## 2. Related Work

Hierarchical federated learning (HFL) has emerged as a promising paradigm for scalable, privacy-preserving, and distributed intrusion detection in IoT environments. Existing studies have explored different aspects of HFL, including architectural design, communication efficiency, robustness, and anomaly detection capabilities.

Saadat et al. [17] proposed an HFL framework for IoT intrusion detection, in which edge servers perform intermediate aggregation to reduce communication overhead. The key contribution of the study is to demonstrate that HFL improves convergence speed and detection accuracy over traditional FL, particularly under non-IID data distributions. Their work enhances scalability and learning efficiency but does not incorporate advanced security or adversarial defense mechanisms. Sun et al. [18] introduced an HFL-based intrusion detection system for 5G-enabled smart grids that integrates a transformer-based detection model. Their novel contribution is the combination of HFL with advanced deep learning architectures to improve detection performance while reducing communication cost. The framework improves accuracy and latency; however, reliance on a single global model limits robustness against adversarial manipulation. Mohawesh et al. [19] proposed an HFL framework that leverages lightweight large language models (LLMs), such as TinyLLaMA and DistilBERT, for B5G IoT environments. The key innovation of the study is the integration of model compression techniques, including knowledge distillation and LoRA, to enable efficient deployment on edge devices. The approach improves scalability and contextual learning capability but does not explicitly address security threats such as poisoning attacks.

Malviya et al. [20] developed an HFL-based intrusion detection framework for IoMT networks, focusing on feature extraction and ensemble learning techniques. Their main contribution is the comparative analysis of linear and non-linear feature extraction methods (PCA, DBN, autoencoders) combined with ensemble classifiers. This enhances detection accuracy and facilitates computational trade-off analysis but lacks mechanisms to handle adversarial behavior and heterogeneous data distributions. Alkulaib et al. [21] proposed an adaptive hierarchical anomaly detection system that combines FL with Growing Hierarchical Self-Organizing Maps (GHSOM). The novel contribution lies in introducing context-aware adaptive hierarchical clustering and selective model updates to reduce communication overhead. The approach improves anomaly-detection efficiency and scalability; however, it remains primarily unsupervised and is not specifically tailored to intrusion-detection classification tasks. Alharbi [22] introduced the AHFL-DAWA framework, an adaptive HFL model with dynamically anomaly-weighted aggregation. The key innovation is the integration of anomaly-driven weighting, differential privacy, and Byzantine fault tolerance with theoretical guarantees. This significantly enhances robustness, scalability, and communication efficiency; however, the framework still depends on a single global model and does not support multi-model personalization.

Elmahfoud et al. [23] investigated the impact of label-flipping attacks on HFL-based intrusion detection systems. Their primary contribution is the systematic analysis of data poisoning vulnerabilities in HFL and the design of a defense mechanism to identify malicious clients. This work enhances understanding of adversarial threats but does not propose fundamentally new aggregation paradigms. Rabieinejad et al. [24] proposed a two-level privacy-preserving FL framework using partially homomorphic encryption for IoT attack detection. The key contribution is the integration of encryption-based secure aggregation to protect model updates without significant performance degradation. The framework enhances privacy and confidentiality, it does not address hierarchical scalability or non-IID learning challenges. In our prior study [12], we proposed a two-tier secure HFL framework that integrates lightweight cryptographic mechanisms to enable secure edge–cloud collaboration. The key contribution is the combination of hierarchical aggregation with efficient encryption techniques to ensure confidentiality, integrity, and scalability while reducing communication overhead. Despite these enhancements, the framework relies on averaging-based aggregation and is tested on a non-IoT dataset in an IID distribution, limiting its effectiveness under extreme non-IID conditions and sophisticated adversarial.

Beyond HFL-based intrusion detection, broader research in FL has explored robustness, aggregation diversity, and personalization. Robust aggregation methods such as Krum and Trimmed Mean aim to mitigate adversarial updates by filtering or clipping outliers, while personalized and clustered FL approaches maintain multiple model representations to address data heterogeneity. Recent surveys [25,26] provide comprehensive taxonomies of these techniques. Unlike these approaches, which typically operate under a single-level FL setting, the proposed framework integrates similarity-aware clustering and multi-model learning within a hierarchical architecture, enabling both robustness and adaptability under extreme non-IID IoT conditions.

Despite the advancements in HFL for IoT intrusion detection, several limitations remain. Most existing approaches rely on single-model aggregation, which is inadequate for handling extreme non-IID data distributions. Additionally, many frameworks adopt averaging-based aggregation, making them vulnerable to stealthy poisoning and backdoor attacks. Although some studies address scalability, privacy, or robustness individually, unified solutions that jointly provide adaptive learning, robust aggregation, and model diversity remain limited.

To address these challenges, this work proposes an adaptive multi-model HFL framework that integrates similarity-aware clustering, multi-model aggregation, and dynamic client-side model selection, thereby improving robustness, adaptability, and performance in realistic IoT intrusion detection scenarios.

## 3. The Proposed AMM-HFL Framework

**Figure 1:**
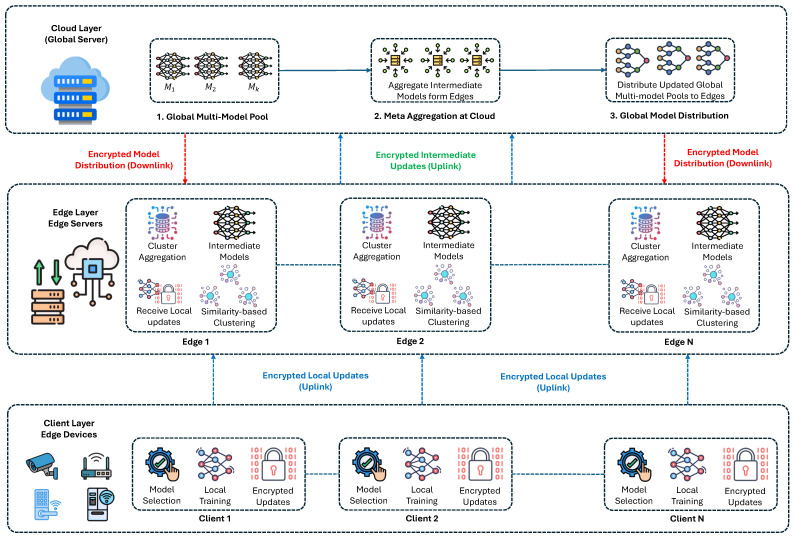
A high-level architectural diagram of the proposed framework.

This section presents the formal model of the proposed AMM-HFL framework for IoT intrusion detection. A high-level workflow of the proposed framework is presented in Figure 1. The framework extends conventional hierarchical FL by integrating similarity-aware aggregation, multi-model learning, and lightweight cryptographic protection within a unified edge–cloud architecture.

### 3.1. System Architecture and Notation

The architecture follows a hierarchical design in which distributed IoT clients collaborate through intermediate edge servers before interacting with the cloud. This structure enables scalable learning while preserving locality and reducing communication overhead.

Consider a distributed IoT system composed of three types of entities: a set of clients $C=\left\{C_{1},C_{2},\ldots ,C_{N}\right\}$, a set of edge (fog) servers $E=\left\{E_{1},E_{2},\ldots ,E_{K}\right\}$, and a central cloud server *S*. Each edge server $E_{k}$ manages a subset of clients $C_{k}\subset C$, such that ${⋃}_{k=1}^{K}C_{k}=C$ and $C_{i}\cap C_{j}=\emptyset$ for i≠j. Let $n_{k}=\left|C_{k}\right|$ denote the number of clients associated with edge server $E_{k}$.

Each client $C_{i}$ possesses a local dataset $D_{i}$ that follows a highly heterogeneous, non-independent distribution. In particular, the system operates under extreme non-IID conditions, where each client observes only a limited subset of attack classes. Let $f_{\theta }$ denote the deep neural network used for intrusion detection, parameterized by $\theta \in R^{d}$. Unlike conventional FL, the global model is represented as a set of candidate models, as defined in (1), where multiple model parameters are maintained to capture heterogeneous data distributions.

(1) $$\Theta^{\left(t\right)}=\left\{\theta_{1}^{\left(t\right)},\theta_{2}^{\left(t\right)},\ldots ,\theta_{M}^{\left(t\right)}\right\},$$

where *M* evolves dynamically across communication rounds.

### 3.2. Secure Key Establishment and Model Protection

This stage ensures secure communication between clients and edge servers, forming the foundation for privacy-preserving collaboration. The objective is to protect model updates during transmission while maintaining computational efficiency suitable for resource-constrained IoT environments.

Secure communication between clients and edge servers is established using elliptic-curve Diffie–Hellman key exchange. Each client–edge pair $\left(C_{i},E_{k}\right)$ generates public–private key pairs and computes a shared secret as shown in (2):

(2) $$s_{ik}=\mathrm{ECDH}\left(sk_{i},pk_{k}\right)=\mathrm{ECDH}\left(sk_{k},pk_{i}\right).$$

A symmetric encryption key is derived using a hash-based key derivation function using (3):

(3) $$K_{ik}=\mathrm{HKDF}\left(s_{ik},\;\mathrm{info}\;\right),$$

where BLAKE2b is used as the underlying hash function. The local model parameters are serialized and encrypted using ChaCha20-Poly1305 as shown in (4):

(4) $${\tilde{\theta }}_{i}^{\left(t\right)}={\mathrm{Enc}}_{K_{ik}}\left(\theta_{i}^{\left(t\right)}\right).$$

**Figure 2:**
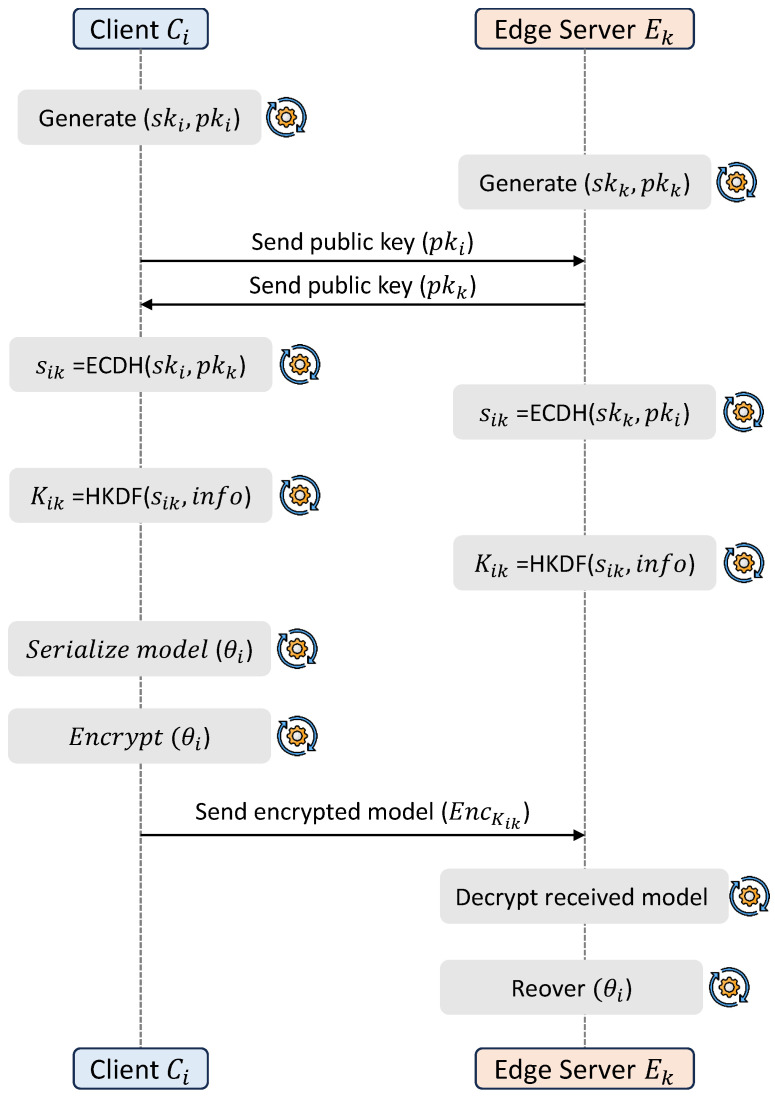
Workflow of secure key establishment and model protection.

This mechanism ensures the confidentiality and integrity of model updates during transmission from clients to edge servers. The secure key establishment and model protection process is illustrated in Figure 2.

### 3.3. Client-Side Training and Model Selection

**Figure 3:**
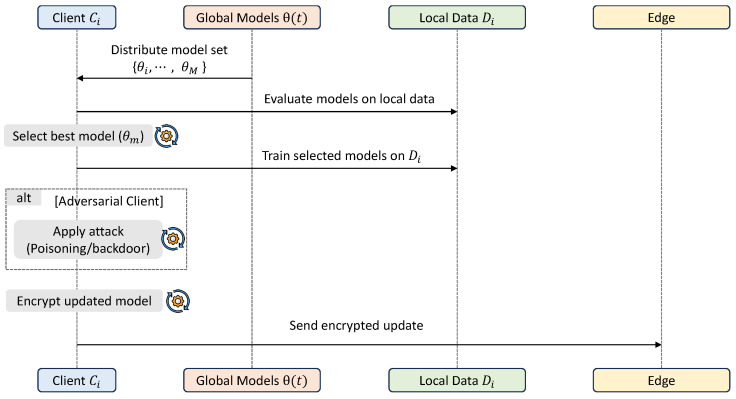
Workflow of client-side training and model selection.

This stage governs local learning on IoT devices and introduces adaptive model selection, enabling each client to align with the most suitable global model under heterogeneous data conditions. This mechanism is critical for handling extreme non-IID distributions and improving personalization. The workflow of this stage is summarized in Figure 3.

At each communication round *t*, each client selects the most suitable model from the available model set $\Theta^{\left(t\right)}$ by minimizing the local empirical loss as presented in (5):

(5) $$m_{i}^{\left(t\right)}=\arg\min_{m\in \{1,\ldots ,M\}}L\left(f_{\theta_{m}^{\left(t\right)}},D_{i}\right)$$

The selected model $\theta_{m_{i}}^{\left(t\right)}$ is used as the initialization for local training. The client then updates the model parameters using stochastic gradient descent (6):

(6) $$\theta_{i}^{\left(t\right)}=\theta_{m_{i}}^{\left(t\right)}-\eta \nabla L\left(f_{\theta_{m_{i}}^{\left(t\right)}},D_{i}\right),$$

where η is the learning rate. To address class imbalance in intrusion detection data, the loss function is weighted according to class frequencies.

Adversarial behavior is modeled by allowing a subset of clients to generate malicious updates. These include naive poisoning through parameter perturbations and stealthy attacks via label manipulation, both of which introduce biased updates while remaining difficult to detect.

### 3.4. Edge-Level Similarity-Aware Aggregation

**Figure 4:**
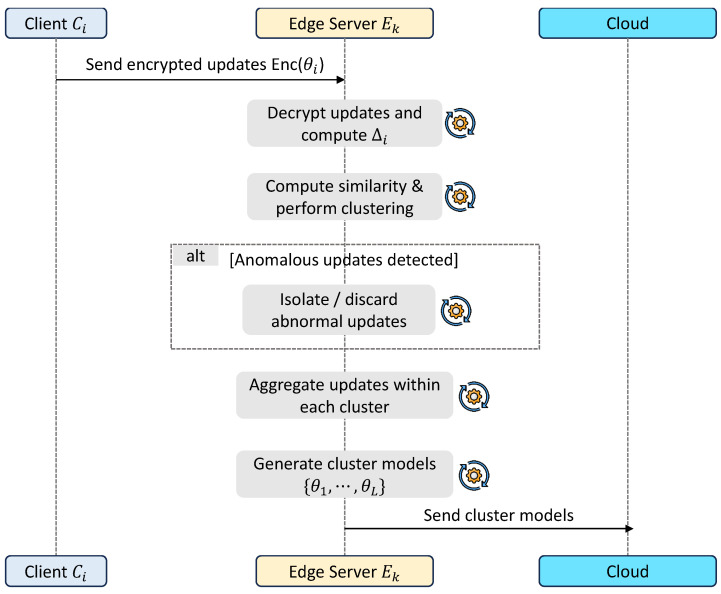
Workflow of edge-level similarity-aware aggregation.

This stage performs robust intermediate aggregation at the edge layer, where the primary objective is to filter, group, and consolidate client updates before forwarding them to the cloud. It plays a central role in improving robustness against adversarial updates while preserving meaningful local patterns. The overall workflow of this stage is presented in Figure 4.

Upon receiving encrypted model updates ${\tilde{\theta }}_{i}^{\left(t\right)}$, each edge server $E_{k}$ decrypts them using the corresponding keys $K_{ik}$ and computes model deviations with respect to the selected base model as shown in (7):

(7) $$\Delta \theta_{i}^{\left(t\right)}=\theta_{i}^{\left(t\right)}-\theta_{m_{i}}^{\left(t\right)}.$$

To ensure consistency of update representations, clustering is performed separately for each base model $\theta_{m}^{\left(t\right)}$. That is, only updates $\Delta \theta_{i}^{\left(t\right)}$ computed with respect to the same selected model $\theta_{m_{i}}^{\left(t\right)}$ are grouped and processed together. This guarantees that all updates within a cluster share a common reference model. To enable robust aggregation, each update is vectorized into $v_{i}^{\left(t\right)}\in R^{d}$, and pairwise cosine similarity is computed using (8):

(8) $$\mathrm{sim}\left(i,j\right)=\frac{v_{i}^{\left(t\right)}\cdot v_{j}^{\left(t\right)}}{∥v_{i}^{\left(t\right)}∥∥v_{j}^{\left(t\right)}∥}$$

A distance matrix is defined as d(i,j)=1−sim(i,j), and hierarchical clustering partitions updates (grouped per base model $\theta_{m}^{\left(t\right)}$) into clusters {G1,…,GL}.

Updates that exhibit abnormal norms or form isolated clusters are identified as anomalous and either excluded or isolated based on statistical thresholds. For each valid cluster $G_{l}$, an aggregated update is computed using (9):

(9) $$\Delta \theta_{l}^{\left(t\right)}=\frac{1}{\left|G_{l}\right|}\sum_{i\in G_{l}}\Delta \theta_{i}^{\left(t\right)},$$

and the corresponding cluster model is obtained as (10):

(10) $$\theta_{l}^{\left(t+1\right)}=\theta_{m}^{\left(t\right)}+\Delta \theta_{l}^{\left(t\right)}.$$

Here, $\theta_{m}^{\left(t\right)}$ denotes the common base model associated with all updates in cluster $G_{l}$.

This process produces multiple intermediate models representing coherent groups of client updates.

### 3.5. Cloud-Level Meta-Aggregation

**Figure 5:**
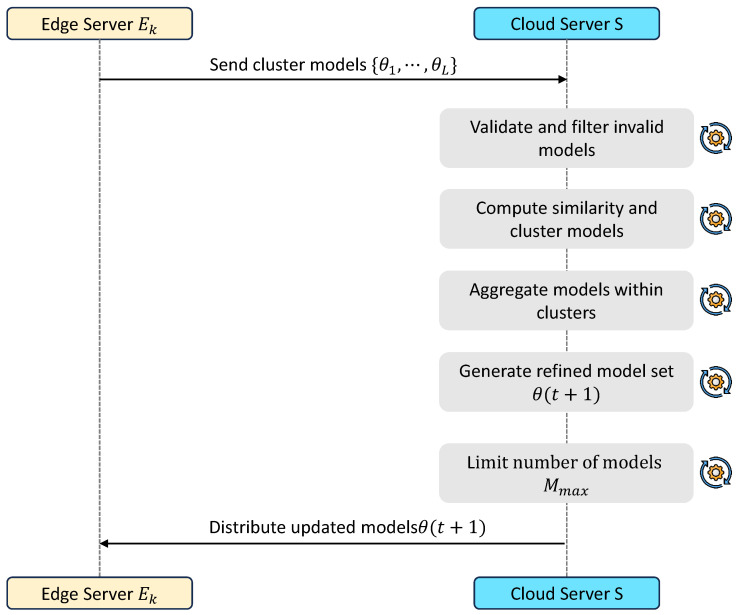
Workflow of cloud-level meta-aggregation.

This stage refines and consolidates the intermediate models generated at the edge layer. The objective is to balance diversity and generalization by merging similar models while maintaining multiple representations to capture heterogeneous data patterns. The cloud-level aggregation process is highlighted in Figure 5.

The cloud server receives a set of intermediate models (11):

(11) $$M^{\left(t+1\right)}=\left\{\theta_{l}^{\left(t+1\right)}\right\}.$$

Similarity-based clustering is again applied to group models with similar characteristics. For each cluster $H_{q}$, a merged model is computed using (12):

(12) $$\theta_{q}^{\left(t+1\right)}=\frac{1}{\left|H_{q}\right|}\sum_{\theta \in H_{q}}\theta .$$

The resulting set $\Theta^{\left(t+1\right)}=\left\{\theta_{q}^{\left(t+1\right)}\right\}$ is constrained to a maximum size $M_{\max}$ to ensure scalability, forming the updated global model set.

### 3.6. Global Inference and Model Utilization

This stage defines how the learned models are utilized for prediction and decision-making. By leveraging multiple specialized models, the framework improves robustness and generalization across diverse intrusion patterns.

Given an input sample *x*, predictions from all models are combined using ensemble averaging (13):

(13) $$\hat{y}=\arg\max\left(\frac{1}{M}\sum_{m=1}^{M}f_{\theta_{m}}\left(x\right)\right)$$

This ensemble mechanism enhances detection performance by aggregating knowledge from multiple model representations.

The proposed framework differs from traditional FL by using a multi-model representation that captures diverse data distributions under highly non-IID conditions. The similarity-aware clustering mechanism at both edge and cloud levels enables robust aggregation by isolating anomalous updates and preserving consistent patterns. In addition, lightweight cryptographic protection ensures secure communication without imposing significant overhead. The integration of these components results in a unified framework capable of addressing scalability, robustness, and security challenges in IoT intrusion detection.

## 4. Experiments and Results

### 4.1. Experimental Setup

The proposed AMM-HFL framework was implemented and evaluated in a Google Colab Pro environment with an NVIDIA L4 GPU using a real-time IoT dataset IDSIoT2024 [27]. The core system architecture was built with the PyTorch 2.10.0+cu128 deep learning framework, enabling the development of a customized multilayer perceptron (MLP). Given the high variance and heterogeneous nature of the dataset, the PyTorch implementation integrated Layer Normalization across deep fully connected layers and paired it with an AdamW optimizer, which serves as the practical instantiation of the gradient-based update described in Equation (6), to ensure robust local convergence.

#### 4.1.1. Hierarchical Federated Learning Topology and Setup

The simulation was structured around the proposed three-tier hierarchical FL topology, comprising the IoT client tier, the edge server tier, and the central cloud tier. To simulate a highly distributed IoT network, the environment was configured with 5 independent edge servers, each managing a dedicated cluster of 10 resource-constrained IoT clients, resulting in a total network size of 50 collaborative client nodes.

The federated training process was executed over 50 rounds of global communication to ensure stable convergence and robustness evaluation under both IID and extreme non-IID conditions, including scenarios with adversarial clients. During each round, individual IoT clients dynamically evaluated the available cloud-provided global models and initialized their training weights based on the lowest local empirical loss. Clients then performed local training for 3 epochs using a batch size of 64 before encrypting and transmitting their updated parameters to their designated edge server. At the intermediate tier, edge servers performed localized, similarity-aware clustering to generate refined intermediate models, which were then transmitted to the central cloud server for final meta-aggregation.

#### 4.1.2. Libraries and Cryptographic Integration

Beyond the core neural network, the implementation leveraged scikit-learn for critical data preprocessing and clustering. Standard scaling and label encoding were applied to the raw traffic data. Scikit-learn’s class weight computation dynamically balanced the highly imbalanced distributions inherent in intrusion detection datasets. Furthermore, clustering was employed at the edge tier to dynamically group the extracted cosine-similarity matrices of the decrypted model updates.

To ensure secure model transmissions across the hierarchy without overwhelming resource-constrained IoT devices, the cryptography Python 43.0.3 package was integrated. We used the X25519 standard [28] for Elliptic-Curve Diffie–Hellman (ECDH) key exchanges and ChaCha20Poly1305 for authenticated symmetric encryption, achieving an optimal balance between cryptographic strength and computational efficiency.

#### 4.1.3. Hyperparameter Configuration

The performance and behavior of the FL environment are governed by a defined set of hyperparameters, carefully tuned to simulate an extreme edge-computing scenario. Table 1 outlines the comprehensive configuration used in the simulation.

#### 4.1.4. Data Partitioning and Adversarial Scenarios

To accurately reflect realistic, highly localized IoT deployments, the dataset was partitioned using two distinct distribution strategies. In the Independent and Identically Distributed (IID) scenario, data shards were allocated uniformly, ensuring that each client had a representative sample of all global traffic classes. Conversely, the Extreme Non-IID scenario was designed to simulate specialized edge sensors. The dataset was sorted by label, and clients were strictly limited to observing up to three specific traffic classes. This artificially induced extreme local bias poses a severe challenge to traditional centralized aggregation methods.

In these environments, adversarial behavior was introduced by designating varying proportions of the network (5%, 15%, and 30%) as compromised. These clients deployed a dual-threat strategy. A portion executed naive model-poisoning attacks by injecting high-magnitude Gaussian noise into locally computed gradients, while others performed stealthy backdoor attacks via label manipulation. By forcing the model to misclassify specific malicious traffic as normal, these attacks introduce subtle vulnerabilities while maintaining low parameter deviations to evade standard statistical detection.

To model realistic intermittent adversarial activity, only this predefined subset of compromised clients generates malicious updates with a fixed probability (0.3) at each communication round, while benign clients always produce legitimate updates.

### 4.2. Model Performance

**Figure 6:**
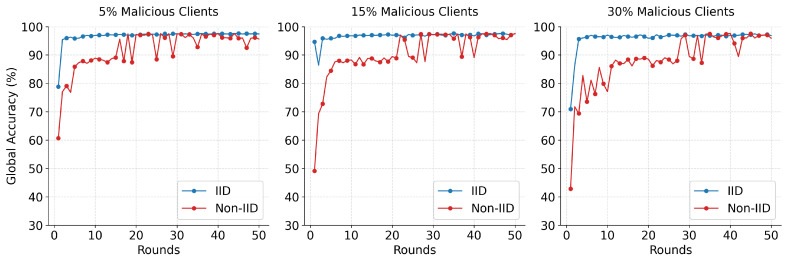
Global model convergence across communication rounds under varying malicious client ratios.

The global convergence and classification capabilities of the AMM-HFL architecture were systematically evaluated under escalating adversarial threat levels. As illustrated in Figure 6, the global ensemble model exhibits rapid initial learning, reaching near-peak accuracy within the first 10 communication rounds across both data distribution strategies. Under IID conditions, the learning trajectory remains stable, settling into a steady state with minimal variance. In contrast, Extreme Non-IID conditions introduce observable early-stage volatility. This fluctuation is a natural mathematical consequence of the highly skewed local data views; however, the similarity-aware clustering mechanism reduces this variance, enabling convergence by approximately the 20th communication round.

**Figure 7:**
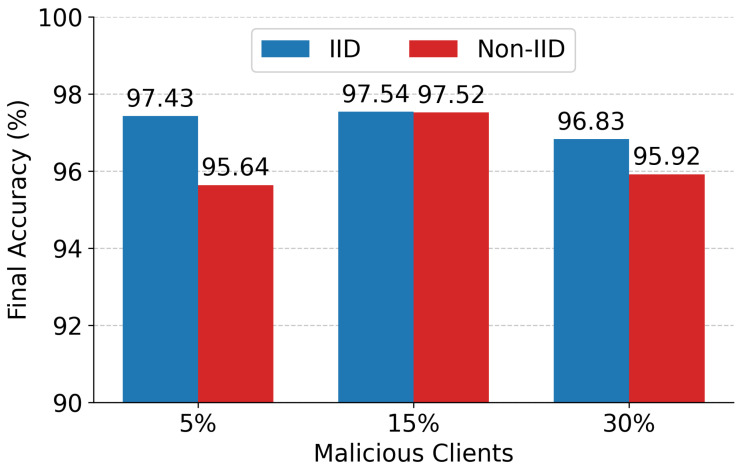
Final Global model accuracy under different scenarios.

Figure 7 highlights the system’s final ensemble accuracy, demonstrating its resilience even under massive network compromise. In the most severe scenario, where 30% of the active client pool is malicious, the IID deployment achieved a final accuracy of 96.83%, while the heavily constrained Non-IID deployment reached 95.92%. This limited degradation (≤1% accuracy drop) indicates that edge-level clustering isolates anomalous updates prior to cloud aggregation.

**Figure 8:**
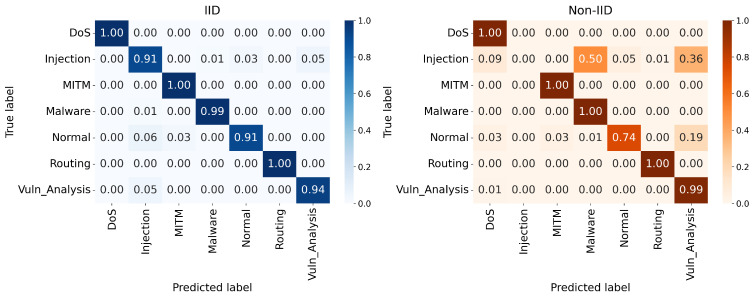
Confusion matrices under 5% malicious clients.

**Figure 9:**
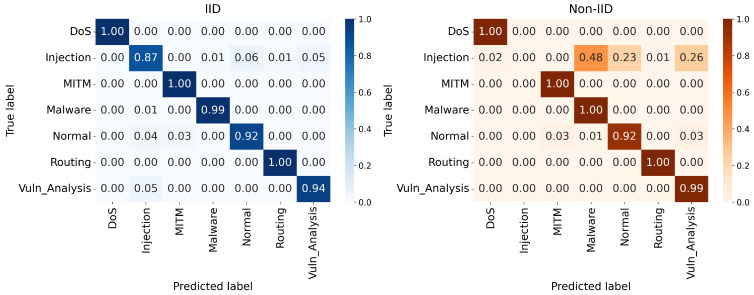
Confusion matrices under 15% malicious clients.

**Figure 10:**
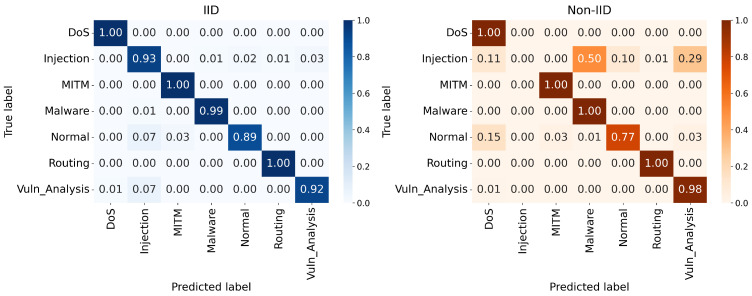
Confusion matrices under 30% malicious clients.

The multiclass performance of the proposed design is further clarified by the confusion matrices shown in Figure 8, Figure 9 and Figure 10. In the IID deployments, the architecture demonstrates near-perfect classification capabilities for critical attack vectors, consistently achieving true positive rates of 0.99 to 1.00 for DoS, MITM, Malware, and Routing attacks. However, the Extreme Non-IID constraint introduces necessary mathematical trade-offs in specific minority classes. As the major attack classes retain exceptional accuracy, the “Injection” class suffers a noticeable reduction in detection precision under non-IID settings, occasionally being misclassified as Normal traffic. This dynamic occurs because strict multi-model aggregation occasionally outvotes specialized clusters when a specific attack pattern is extremely rare among localized IoT nodes.

### 4.3. Computational Performance

**Figure 11:**
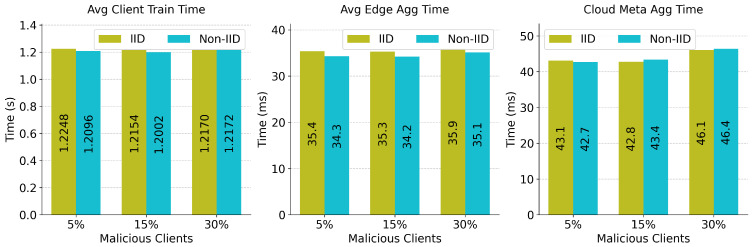
Average latency profile across client, edge, and cloud tiers for IID and Non-IID deployments.

For an intrusion detection framework to be viable in real-world IoT networks, it must meet stringent latency constraints. The computational performance was profiled across the three architectural tiers, as shown in Figure 11.

The latency analysis shows that the local client-side training phase is the most time-consuming operation, consuming an average of 1.20 to 1.22 s per communication round. Once encrypted updates reach the intermediate edge servers, secure decryption, model deviation derivation, cosine similarity computation, and subsequent clustering execute efficiently, requiring only 34.2 ms to 35.9 ms on average. The final cloud-tier meta-aggregation is similarly lightweight, operating in approximately 42.7 ms to 46.4 ms. Notably, the processing overhead remains virtually identical whether the system manages an IID or a highly fragmented Non-IID distribution, demonstrating that the similarity-aware hierarchical routing scales exceptionally well without introducing unpredictable processing bottlenecks.

### 4.4. Security Evaluation

**Figure 12:**
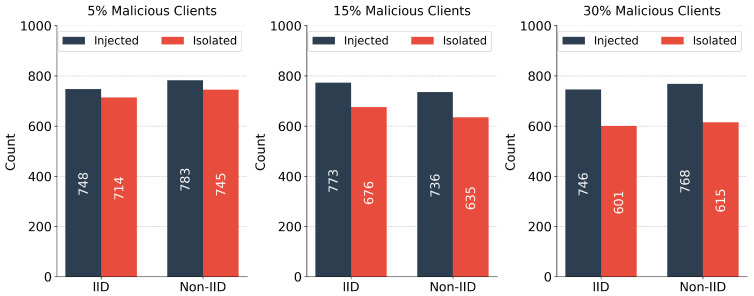
Proactive fog security defense illustrating the cumulative number of adversarial model updates injected and isolated across all communication rounds.

The primary defense mechanism of the AMM-HFL architecture relies on proactive, edge-level statistical isolation. Figure 12 illustrates the direct correlation between the cumulative number of adversarial model updates injected across all communication rounds and the number of those updates that the edge servers successfully identify and discard.

The evaluation shows a high filtering rate, particularly in structurally balanced environments. For instance, in the 5% malicious IID scenario, the edge servers isolated 714 of the 748 injected malicious updates. Stealthy backdoor attacks, intentionally engineered to keep parameter deviations low, occasionally bypass the initial static thresholding. However, the secondary cosine-similarity clustering segregates these divergent models into isolated groups, preventing them from influencing the primary legitimate clusters. As the adversarial presence increases to 30% in the Non-IID scenario, the isolation rate declines slightly. This is because the substantial natural variance in legitimate Non-IID updates makes it mathematically challenging to distinguish a stealthy anomaly from a naturally skewed data shard. Despite this, the downstream ensemble mechanism maintains final accuracy above 95% in the presence of residual noise.

**Figure 13:**
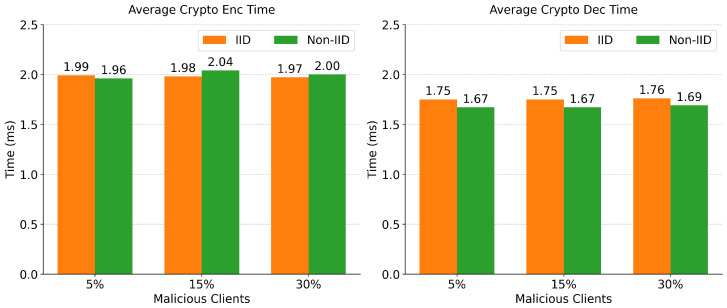
Cryptographic overhead analysis detailing average encryption and decryption latencies.

Figure 13 validates the operational viability of the lightweight cryptography module. Across all experimental conditions, the average time to serialize and encrypt a deep learning model using the ChaCha20Poly1305 cipher remains strictly under 2.04 ms. The corresponding decryption operation at the edge server is similarly efficient, requiring only 1.67 ms to 1.76 ms. When contextualized against the overall client training time, this cryptographic security overhead accounts for less than 0.2% of the total processing pipeline. This indicates that robust data privacy and protection against man-in-the-middle interception can be seamlessly integrated into IoT intrusion detection without degrading the system’s computational efficiency.

### 4.5. Resilience Against Advanced Adaptive Attacks

To validate the AMM-HFL framework’s resilience against highly sophisticated adversaries, a supplementary security evaluation was conducted that goes beyond standard noise injection and naive backdoors. This evaluation introduced three advanced threat models explicitly designed to exploit and bypass FL defenses. Specifically, it tested optimization-based stealthy attacks in which adversaries apply an L2 regularization penalty during local training to artificially minimize parameter distance and evade norm-based anomaly detection. It also evaluated aggressive model replacement attacks, which use large scaling factors on poisoned weights to mathematically override the global aggregation process, as well as colluding Sybil attacks, in which multiple compromised nodes within a single edge cluster synchronize identical malicious updates to form an artificial consensus intended to trick agglomerative clustering metrics.

**Figure 14:**
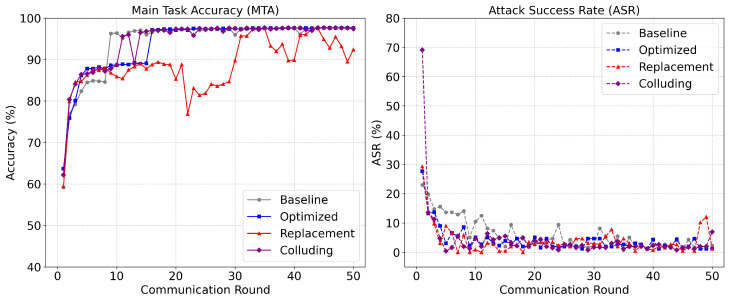
Adaptive Multi-Model HFL resilience against advanced adversaries.

As illustrated in Figure 14, the proposed framework mitigates these advanced threats over 50 communication rounds without compromising the system’s primary intrusion detection capabilities. The Main Task Accuracy (MTA) remains robust, tracking closely with the baseline for optimized and colluding attacks and rapidly recovering to above 90% even after the severe mathematical perturbations induced by the model replacement attack between rounds 10 and 30. More importantly, the Attack Success Rate (ASR) validates the efficacy of the hierarchical defense mechanisms. After a brief initial vulnerability period before statistical baselines are firmly established, the system suppresses the ASR to near-zero levels starting in round 5. By successfully identifying zero-variance Sybil clones, clipping excessive update magnitudes, and isolating negatively correlated stealth parameters, the AMM-HFL framework demonstrates consistent robustness against adaptive adversaries under the evaluated conditions.

### 4.6. Performance Comparison with Aggregation Baselines

**Figure 15:**
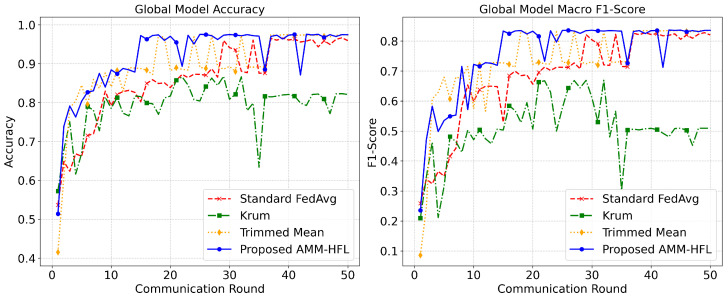
Aggregation strategy comparison under extreme Non-IID + 30% attackers.

To rigorously validate the robustness of the proposed architecture, a comparative analysis was conducted against standard and state-of-the-art robust aggregation baselines. These implementations include Standard FedAvg, Krum, and Trimmed Mean under an extreme non-IID distribution with a 30% ratio of malicious clients. As illustrated in Figure 15, conventional single-model aggregation paradigms exhibit distinct vulnerabilities in this highly heterogeneous and adversarial environment. Standard FedAvg demonstrates significant variance and instability across communication rounds, as its naive averaging mechanism incorporates injected noise and backdoor manipulations. Krum struggles the most, exhibiting the lowest accuracy and F1-scores by a wide margin. Because it assumes an IID distribution to filter outliers, it frequently misidentifies highly specialized, legitimate minority-class updates as anomalous and discards them. Trimmed Mean offers much better resilience by clipping statistical extremes, allowing it to achieve performance closer to the proposed method in later rounds, yet it still suffers from noticeable instability and sharp dips throughout the training process.

In contrast, the proposed AMM-HFL consistently achieves higher performance than the evaluated baselines under the considered settings. As evidenced by the top-performing trajectory in Figure 15, AMM-HFL rapidly converges to superior Accuracy and Macro F1-Scores, maintaining them with minimal variance. By abandoning the restrictive single-model constraint in favor of similarity-aware clustering, AMM-HFL dynamically separates conflicting updates at the edge. This mechanism isolates adversarial parameter manipulations in discarded outlier clusters while aggregating divergent legitimate updates into a refined set of multi-model representations. Consequently, the framework preserves highly skewed local data patterns without compromising global security, ensuring sustained robustness even when nearly a third of the participating edge nodes are compromised.

### 4.7. Ablation Study

**Figure 16:**
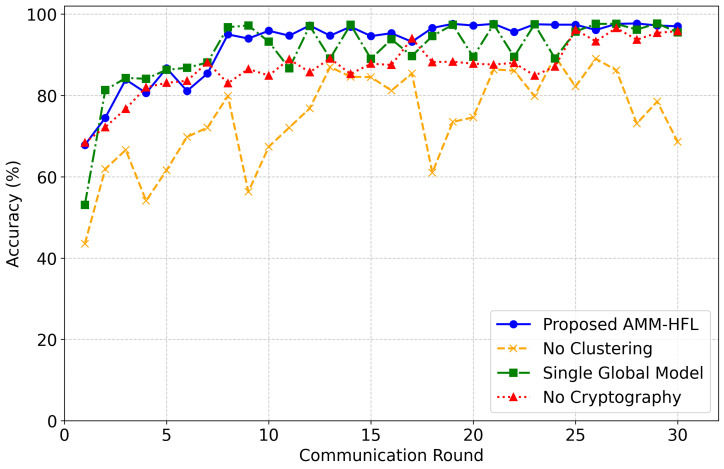
Experimental outcomes of ablation study.

To systematically quantify the contributions of the framework’s core architectural components, an ablation study was conducted under extreme non-IID conditions, with a 15% ratio of malicious clients. As shown in Figure 16, disabling edge-tier similarity clustering fundamentally degrades the system’s defensive posture. Without this vital filtering mechanism, unmitigated adversarial updates trigger severe accuracy collapses and extreme instability across all communication rounds. Furthermore, retaining clustering while forcing the cloud tier to consolidate updates into a Single Global Model results in violent oscillations in accuracy. This erratic trajectory empirically demonstrates that a single model representation is mathematically inadequate for reliably capturing and retaining the highly divergent, skewed traffic patterns inherent in specialized IoT edge sensors.

The evaluation also tested a variant bypassing the encryption modules to assess the impact of the security layer. While this variant avoids the catastrophic failures observed in the structural ablations, it unexpectedly exhibits a slower, slightly lower convergence trajectory during intermediate learning phases compared to the fully intact architecture, while completely sacrificing critical data confidentiality guarantees. Ultimately, the fully integrated AMM-HFL framework demonstrates superior performance, rapidly converging to a highly stable, peak accuracy trajectory. These results demonstrate that synergy among similarity-aware clustering, multi-model generalization, and cryptographic protection is essential for robust, secure, and consistent intrusion detection.

### 4.8. Performance Comparison with Related Work

Table 2 presents a comparative analysis of the proposed AMM-HFL framework against state-of-the-art HFL-based intrusion detection systems. It is important to note that Table 2 provides a contextual comparison rather than a direct benchmark, as existing HFL-based IDS studies are evaluated on different datasets, metrics, and experimental settings. In contrast, the proposed framework is evaluated on the recent IDSIoT2024 dataset, which better reflects realistic IoT traffic and adversarial conditions.

Existing approaches primarily rely on single-model aggregation strategies, such as averaging or weighted aggregation, and often address specific aspects such as scalability, privacy, or robustness in isolation. For instance, Refs. [17,18] employ conventional averaging-based HFL, achieving moderate-to-high performance but lacking explicit security mechanisms. Similarly, Refs. [19,20] enhance detection performance through advanced models and feature engineering; however, they do not incorporate defenses against adversarial attacks. More recent frameworks, such as [22], introduce robustness through anomaly-weighted aggregation and differential privacy, while [24] focuses on secure aggregation via homomorphic encryption. Although these methods improve specific dimensions, they still rely on single global models and remain limited in handling extreme non-IID data distributions.

In contrast, the proposed AMM-HFL framework integrates similarity-aware clustering, multi-model aggregation, and lightweight cryptographic protection within a unified architecture. Our design enables effective isolation of adversarial updates while preserving model diversity, leading to superior performance under both IID and extreme non-IID settings. The proposed framework achieves 96.83% to 97.54% accuracy under IID conditions and 95.64% to 97.52% under non-IID conditions, outperforming or matching existing methods while providing enhanced robustness and adaptability.

## 5. Conclusions

This paper presented an adaptive multi-model hierarchical federated learning framework for robust and scalable IoT intrusion detection in highly heterogeneous and adversarial environments. By combining similarity-aware clustering at the edge layer with meta-aggregation at the cloud layer, the proposed approach goes beyond conventional averaging-based FL and enables the generation of multiple specialized models that better capture diverse local data distributions. The incorporation of lightweight cryptographic mechanisms ensures secure communication while maintaining efficiency for resource-constrained IoT devices. Extensive experimental evaluation under both IID and extreme non-IID settings, including scenarios with a significant proportion of malicious clients, demonstrates that the framework achieves strong detection performance and reduces the impact of poisoning and backdoor attacks under these conditions. These results emphasize the value of adaptive aggregation and model diversity in enhancing the robustness and scalability of federated intrusion detection systems.

A promising direction for future work is to extend the proposed framework to support real-time streaming data and evolving attack patterns. This would enable the model to adapt continuously to concept drift in dynamic IoT environments, thereby improving its practical applicability and long-term effectiveness in real-world deployments.

## Figures and Tables

**Table 1:** Comprehensive simulation hyperparameters.

Parameter	Value	Description
Total IoT Clients	50	Total number of distributed devices in the FL network.
Edge Servers	5	Intermediate aggregation nodes.
Global Communication Rounds	50	Total iterations of the global federated training process.
Client Local Epochs	3	Full passes over local data per client before edge aggregation.
Batch Size	64	Number of tabular samples processed per local gradient update.
Learning Rate	0.001	Step size for the AdamW optimizer.
Extreme Non-IID Classes	3	Maximum distinct attack classes visible per client.
Cosine Distance Threshold	0.4	Boundary for isolating anomalous update vectors via clustering.
Attack Probability	0.3	Baseline likelihood of a client exhibiting malicious behavior.
Malicious Client Ratio	5%, 15%, 30%	Proportion of the client pool executing adversarial actions.
Model Poisoning Strength	0.5	Gaussian noise multiplier applied during naive Byzantine attacks.

**Table 2:** Performance comparison with state-of-the-art HFL-based IDS studies.

Ref.	Proposed Approach	Data Distribution	Security Characteristics	Aggregation Strategy	Performance Score
[17]	HFL-based IDS	IID/Non-IID	Not addressed	Averaging	∼78% (IID), ∼77% (non-IID)
[18]	HFed-IDS (Transformer-IDM)	Not specified	Not addressed	Averaging	95%
[19]	HFL + Lightweight LLMs	Non-IID	Not addressed	Averaging	96.2% (F1 Score)
[20]	HFL + Feature Extraction + Ensemble	Not specified	Not addressed	Central aggregation	97.96%
[21]	FL + Adaptive GHSOM	Not specified	Not addressed	Selective updates	94% (Precision)
[22]	AHFL-DAWA	Non-IID	Differential Privacy + Byzantine Robustness	Weighted aggregation	96.8% (IID), 94.3% (non-IID)
[23]	HFL under Label-Flipping Attacks	IID	Attack detection (poisoning defense)	Averaging + defense	∼95%
[24]	Two-Level FL + Encryption	Not specified	Homomorphic Encryption	Averaging	92.14%, 98.97
[12]	Secure Two-Tier HFL	Non-IID	Lightweight Cryptography + Poisoning Resistance	Averaging	96.04%
Proposed	AMM-HFL (This Work)	Extreme Non-IID	Clustering-based anomaly isolation + implicit poisoning defense + cryptography	Similarity-aware multi-model aggregation	96.83% to 97.54% (IID), 95.64% to 97.52% (non-IID)

## Data Availability

The experimental evaluation in this study was conducted exclusively using the publicly available IDSIoT2024 dataset, and no new data were generated. The processed data and Jupyter Notebooks (Version 6.5.7) supporting the reproduction of the proposed framework are available from the authors upon reasonable request for research purposes.

## References

[1] Khan M.A., Khan Khattk M.A., Latif S., Shah A.A., Ur Rehman M., Boulila W., Driss M., Ahmad J. (2021). Voting classifier-based intrusion detection for iot networks. Advances on Smart and Soft Computing: Proceedings of ICACIn 2021, Casablanca, Morocco, 24–25 May 2021.

[2] Kołaczek G. (2025). Internet of things (iot) technologies in cybersecurity: Challenges and opportunities. Appl. Sci..

[3] Latif S., Driss M., Boulila W., Huma Z.E., Jamal S.S., Idrees Z., Ahmad J. (2021). Deep learning for the industrial internet of things (iiot): A comprehensive survey of techniques, implementation frameworks, potential applications, and future directions. Sensors.

[4] Abualghanam O., Alazzam H., Almobaideen W. (2025). Hierarchical lightweight intrusion detection system using deep learning in the context of IoT. Clust. Comput..

[5] Zhukabayeva T., Zholshiyeva L., Karabayev N., Khan S., Alnazzawi N. (2025). Cybersecurity solutions for industrial internet of things–edge computing integration: Challenges, threats, and future directions. Sensors.

[6] Huma Z.E., Jan S.U., Ahmad J., Buchanan W., Pitropakis N. (2026). Adversarial machine learning in IoT security: A comprehensive survey. ACM Comput. Surv..

[7] Laidi R., Merabtine N., Djenouri D., Latif S., Qadir H.A., Djenouri Y., Balasingham I. (2025). Federated learning in IoT environments: Examining the three-way see-saw for privacy, model-performance, and network-efficiency. IEEE Commun. Surv. Tutor..

[8] Dubey P., Kumar M. (2025). Integrating Explainable AI with Federated Learning for Next-Generation IoT: A comprehensive review and prospective insights. Comput. Sci. Rev..

[9] Latif S., Djenouri D., Adamatzky A. (2025). An Integrated Approach to Mitigate Poisoning Attacks in Federated Learning Frameworks. Proceedings of the 2025 International Joint Conference on Neural Networks (IJCNN), Rome, Italy, 30 June–5 July 2025.

[10] Alqattan D.S., Snasel V., Ranjan R., Ojha V. (2025). Analysis of deep learning under adversarial attacks in hierarchical federated learning. High-Confid. Comput..

[11] Kumar K.N., Mohan C.K., Cenkeramaddi L.R. (2023). The impact of adversarial attacks on federated learning: A survey. IEEE Trans. Pattern Anal. Mach. Intell..

[12] Latif S., Djenouri D. (2025). A Two-Tier Secure Federated Learning Framework with Lightweight Cryptography for Edge-Cloud Collaboration. Proceedings of the 2025 IEEE 35th International Telecommunication Networks and Applications Conference (ITNAC), Christchurch, New Zealand, 26–28 November 2025.

[13] Lu Z., Pan H., Dai Y., Si X., Zhang Y. (2024). Federated learning with non-iid data: A survey. IEEE Internet Things J..

[14] Wang Z., Zhu Y., Wang D., Han Z. (2022). Federated analytics informed distributed industrial IoT learning with non-IID data. IEEE Trans. Netw. Sci. Eng..

[15] Arbaoui M., Brahmia M.e.A., Rahmoun A., Zghal M. (2024). Federated learning survey: A multi-level taxonomy of aggregation techniques, experimental insights, and future frontiers. ACM Trans. Intell. Syst. Technol..

[16] AbuAlghanam O., Alazzam H., Almobaideen W., Saadeh M., Saadeh H. (2025). A Novel Key Distribution for Mobile Patient Authentication Inspired by the Federated Learning Concept and Based on the Diffie–Hellman Elliptic Curve. Sensors.

[17] Saadat H., Aboumadi A., Mohamed A., Erbad A., Guizani M. (2021). Hierarchical federated learning for collaborative IDS in IoT applications. Proceedings of the 2021 10th Mediterranean Conference on Embedded Computing (MECO), Budva, Montenegro, 7–10 June 2021.

[18] Sun X., Tang Z., Du M., Deng C., Lin W., Chen J., Qi Q., Zheng H. (2022). A hierarchical federated learning-based intrusion detection system for 5g smart grids. Electronics.

[19] Mohawesh R., Al-Obiedollah H., Maqsood S., Bany Salameh H. (2026). Lightweight large language model based hierarchical federated learning for B5G enabled IoT intrusion detection networks. Clust. Comput..

[20] Malviya A., Singal G. (2025). Hierarchical Federated Learning based Architecture for IDS in IoMT Network. Proceedings of the 2025 IEEE International Conference on Advanced Networks and Telecommunications Systems (ANTS), New Delhi, India, 15–18 December 2025.

[21] Alkulaib L. (2024). Adaptive hierarchical GHSOM with federated learning for context-aware anomaly detection in IoT networks. Proceedings of the 2024 IEEE International Conference on Big Data (BigData), Washington, DC, USA, 15–18 December 2024.

[22] Alharbi F. (2025). Adaptive Hierarchical Federated Learning for IoT Anomaly Detection. IEEE Access.

[23] Elmahfoud E., El Hajla S., Maleh Y., Mounir S., Ouazzane K. (2025). Label flipping attacks in hierarchical federated learning for intrusion detection in IoT. Inf. Secur. J. Glob. Perspect..

[24] Rabieinejad E., Yazdinejad A., Dehghantanha A., Srivastava G. (2024). Two-level privacy-preserving framework: Federated learning for attack detection in the consumer Internet of Things. IEEE Trans. Consum. Electron..

[25] Yin X., Zhu Y., Hu J. (2021). A comprehensive survey of privacy-preserving federated learning: A taxonomy, review, and future directions. ACM Comput. Surv. (CSUR).

[26] Caruccio L., Cimino G., Deufemia V., Iuliano G., Stanzione R. (2024). Surveying federated learning approaches through a multi-criteria categorization. Multimed. Tools Appl..

[27] Koppula M., Joseph L.L. (2025). A real-world dataset “IDSIoT2024” for machine learning/deep learning based cyber attack detection system for IoT architecture. Proceedings of the 2025 3rd International Conference on Intelligent Data Communication Technologies and Internet of Things (IDCIoT), Bengaluru, India, 5–7 February 2025.

[28] Langley A., Hamburg M., Turner S. (2016). RFC 7748: Elliptic Curves for Security. Request for Comments: 7748. https://www.rfc-editor.org/rfc/rfc7748.

